# Successful pregnancy for primary amenorrhea and recurrent implantation failure and the role of hysteroscopic adhesiolysis: a case report

**DOI:** 10.1186/s13256-019-2247-9

**Published:** 2019-10-29

**Authors:** Zakwan Khrait

**Affiliations:** Reproductive Medicine Institute, Dubai, United Arab Emirates

**Keywords:** Hysteroscopy, *In vitro* fertilization, Infertility, Hystero contrast sonography, Recurrent *in vitro* fertilization failure

## Abstract

**Background:**

Infertility continues to be an enigmatic and emerging problem. Although *in vitro* fertilization has proved to be revolutionary and immensely beneficial to many people, it is far from perfect, and many women experience recurrent *in vitro* fertilization failures. There can be a multitude of factors involved in recurrent *in vitro* fertilization failures. The aim of this report was to explore the role of hysteroscopy in determining potential causes of *in vitro* fertilization failure and how the relevant hysteroscopic findings can address the issue of infertility in terms of a subsequent successful *in vitro* fertilization.

**Case presentation:**

A 37-year-old white Arab woman with a history of eight *in vitro* fertilization failures and one curettage performed for a blighted ovum presented to our hospital because of inability to conceive. Her past medical history was significant for hypothyroidism and positive factor V Leiden. She underwent hystero contrast sonography, which revealed a normal uterine cavity with irregular fillings in the right corner. To explore this further, hysteroscopy was performed, which showed dense adhesions in the right upper corner and first-degree adhesions in the lower half of the uterus. After undergoing adhesiolysis and a cycle of estradiol valerate and progesterone, the patient successfully conceived twins.

**Conclusions:**

Hysteroscopy may play an important role before or in conjunction with assisted reproductive techniques to help infertile women and couples achieve their goals of pregnancy and live birth of a child.

## Introduction

The fields of *in vitro* fertilization (IVF) and intracytoplasmic sperm injection (ICSI) have seen significant advances over recent years; however, the implantation rate per embryo transferred usually fails to exceed 30%. IVF failure is caused by multiple factors, including but not limited to the patient’s lifestyle, immune factors, endocrinologic factors, anatomic abnormalities of the female genitalia, and thrombophilia, that can also lead to recurrent IVF failure [1].

The basic workup for evaluation of the uterine cavity consists of transvaginal sonography (TVS) with or without the use of saline or gel as contrast media, possibly followed by either hysterosalpingography (HSG) or hysteroscopy to directly assess the uterine cavity. TVS, as well as saline infusion sonography and gel infusion sonography, are inexpensive and noninvasive and have been shown to be excellent diagnostic tools to detect subtle intrauterine abnormalities [2]. Office hysteroscopy is increasingly recommended as a routine component of the infertility workup [3–5]. It can easily be performed as an outpatient procedure without anesthesia. Moreover, it offers direct visualization and enables clinicians to diagnose and treat intrauterine pathology during the same session [6].

## Case presentation

### History

A 37-year-old white Arab woman with a past history of eight failed IVF cycles presented to our hospital because of inability to conceive for the last 8 years. She was in her normal state of health. She has a history of hypogonadotropic hypogonadism primary amenorrhea, with her menses observed only after Progyluton® (Bayer, Whippany, NJ, USA) administration. She also has a history of hypothyroidism, for which she is currently taking Euthyrox® 50 μg/day (Merck, Darmstadt, Germany).

The patient reported experiencing IVF treatment failure eight times consecutively with a history of recurrent implantation failures. Fresh embryo transfers had been used in all of the previous attempts, with no success. The first IVF attempted resulted in a blighted ovum requiring curettage (dilation and curettage).

Her spouse has also had a semen analysis done, which showed severe oligoasthenoteratospermia, with a sperm count of only 100,000/ml and motility of only 2%. Her family history was nonsignificant except for a history of hypertension in her father. She denied smoking and the use of alcohol or any illicit drug. On physical examination, she was found to have normal development of secondary sexual characteristics, including breast development and hair pattern. Results of her bimanual and rectovaginal examinations were unremarkable.

### Investigations

Results of the patient’s laboratory investigations are shown in Table 1. The patient’s past hormone profile is shown in Table 2.

**Table 1:** Laboratory test results

Test	Value
Cardiolipin antibodies IgM	2.0 mpl/ml; negative
Cardiolipin antibodies IgG	1.9 mpl/ml; negative
Anti-thrombin III, plasma	100%; normal
Lupus anticoagulant	38.4; normal
Protein C	92%; normal
Activated protein C resistance (factor V Leiden)	3.78; positive

*IgG* Immunoglobulin G, *IgM* Immunoglobulin M

**Table 2:** Hormone profile

Hormone	Value
FSH	3.11 IU/L
LH	2.02 IU/L
PROL	25 ng/ml
TSH	2.67 mIU/L
AMH	0.56 ng/ml
E2	116 pmol/L
P4	0.59 ng/ml

*Abbreviations: AMH* Anti-müllerian hormone, *E2* Estradiol, *FSH* Follicle-stimulating hormone, *LH* Luteinizing hormone, *P4* Progesterone, *PROL* Prolactin, *TSH* Thyroid-stimulating hormone

**Fig. 1:**
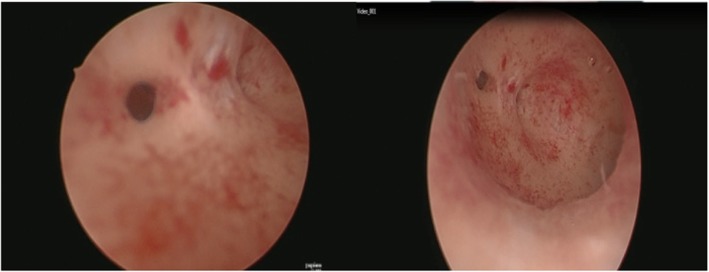
Hysteroscopic findings. Right ostia is seen with synechiae

Hysteroscopy was performed on the patient in July 2015 in India. This procedure revealed a normal uterine cavity with right ostia visualized with synechiae (Fig. 1), whereas the left ostia were seen clearly. No intervention was done at that time.

On presentation at that facility, she was requested to undergo molecular genetic diagnostic (*MTHFR* C677T) gene mutation testing by real-time polymerase chain reaction, and the results revealed the patient to be heterozygous for *MTHFR* C677T gene mutation.

The patient underwent hystero contrast sonography (“HyCoSy”), which showed a normal uterine cavity with irregular filling in the right corner. To explore this ambiguous finding further, hysteroscopy was done in May 2016, which revealed dense adhesions in the right upper corner of the cavity in the fundal area and first-degree adhesions in the lower half of the uterus, which were subsequently removed with a dilator and scope on entry. The final diagnosis was infertility secondary to uterine factors and male factor. No cultural, linguistic, or financial challenges were faced during any phase of managing the patient.

### Management

The patient received one cycle of estradiol valerate and progesterone, and an IVF/ICSI cycle was initiated using an antagonist protocol with the drug Menopur® (Ferring Pharmaceuticals, Parsippany, NJ, USA) 400 IU/day, after which eight oocytes were retrieved. ICSI resulted in three grade 2 embryos, which were transferred on day 3. Luteal support was achieved via progesterone administered orally and vaginally, Clexane (Sanofi, Reading, UK) 40 U given subcutaneously along with prednisolone 5 mg twice daily, and aspirin 75 mg.

**Fig. 2:**
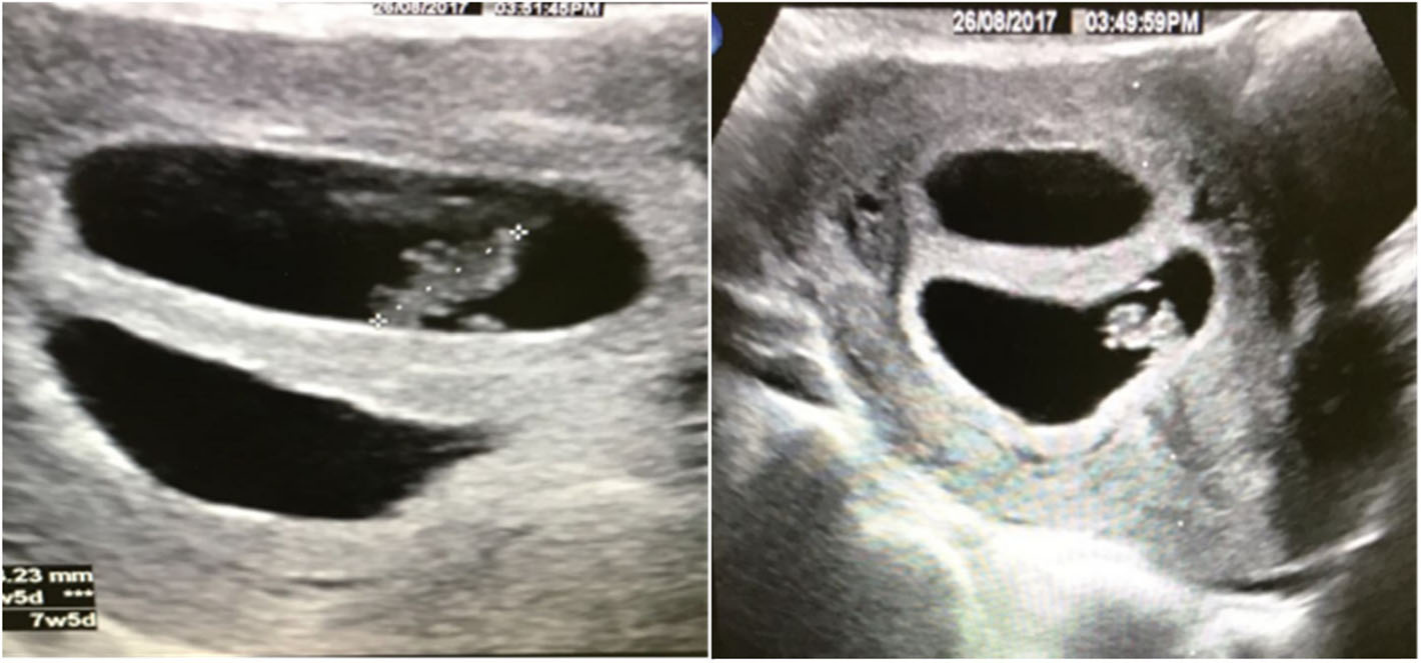
Obstetric scan showing twins with fetal heart positive (6 weeks + 3 days)

The patient was scheduled for follow-up 2 weeks after embryo transfer, and a β-human chorionic gonadotropin test was performed, the result of which became positive (value = 358). The patient was asked to continue prednisolone 5 mg, Clexane 40 U, aspirin 75 mg, and Duphaston (Abbott Healthcare Products, Weesp, The Netherlands). At a subsequent follow-up visit, obstetric ultrasound was performed, which showed twins with a positive fetal heart rate (Fig. 2). Follow-up and delivery were performed at the same hospital at 36 weeks of gestation. Cesarean section was performed to deliver healthy female twins.

### Timeline

A timeline of the patient’s clinical course is provided in Table 3.

**Table 3:** Timeline of the patient’s clinical course

Event	Date
Hysteroscopy performed in India	July 2015
Patient presented to our hospital	March 2016
Hysteroscopy performed by us	May 2016
Management initiated	May 2016
Positive pregnancy test obtained	June 2017

## Discussion

It is widely accepted that a complete infertility workup should include an evaluation of the uterine cavity. Uterine abnormalities, both congenital and acquired, are implicated as one of the most common causes of infertility. In fact, infertility related to uterine cavity abnormalities has been estimated to be the causal factor in as many as 10–15% of couples seeking infertility treatment. Acquired uterine lesions, such as uterine fibroids, endometrial polyps, intrauterine adhesions, or all of these, may cause infertility by interfering with proper embryo implantation and growth. Congenital uterine malformations are also thought to play a role in delaying natural conception. Moreover, abnormal uterine findings have been found in 34–62% of infertile women [7].

The introduction of hysteroscopy in gynecologic practice revolutionized the diagnosis and treatment of intrauterine disease. New methodological and technological developments have made diagnostic and operative hysteroscopy much more efficient, cost-effective, safe, and useful. The most common indication for hysteroscopy is abnormal uterine bleeding, but hysteroscopy is also employed in cases of infertility and Müllerian anomalies [8]. Consequently, hysteroscopy is regarded to be the gold standard modality for uterine cavity exploration.

A review of the effectiveness of hysteroscopy in improving pregnancy rates in subfertile women without other gynecological symptoms concluded that evidence to support the widespread use of hysteroscopic surgery in the general subfertile population was minimal [9]. According to the American Society for Reproductive Medicine, hysteroscopy is the definitive method for the diagnosis and treatment of intrauterine pathology. It is also recommended for further evaluation and treatment of abnormalities defined by less invasive methods, such as HSG and sonohysterography [9].

An assessment of prior studies shows that more than one-third of the patients interpreted as normal following HSG are found to have a uterine abnormality after diagnostic hysteroscopy, which might be a significant cause of reproductive failure. These women may be wrongly treated or unnecessarily investigated as a result of their intrauterine lesion being missed. Treatments given for some abnormalities are known to be beneficial in infertile women, including intrauterine adhesions, congenital uterine malformations, endometrial polyps, and uterine myoma. Chronic endometrial inflammation and micropolyps have also been related to infertility and recurrent miscarriages [10].

In the present case, hysteroscopy results showed dense adhesions on the right upper corner of the cavity on the fundal area and first-degree adhesions on the lower half of the uterus that were removed with a dilator and scope on entry. These adhesions can predispose to primary infertility by not only affecting sperm transportation to the oocyte for fertilization but also preventing implantation of the fertilized ovum even if fertilization occurs, owing to distortion of the uterine cavity. The fact that these findings were detected by hysteroscopy reaffirms the importance of hysteroscopy in the management of infertility. Addressing these findings would therefore improve the chances of the patient conceiving, as observed in the present case.

When debating the need for routine diagnostic hysteroscopy in the evaluation of infertile women, one must keep in mind that hysteroscopy today is no longer a complicated procedure. It not only is simple and fast but also can be performed as an outpatient procedure, requiring short-term training and providing high success rates. Diagnostic hysteroscopy allows complete and accurate identification of intrauterine abnormalities that might negatively affect endometrial receptivity and implantation. The information derived from hysteroscopy helps the physician to institute appropriate therapy, and by doing so, it improves conception rates over shorter intervals.

A study was done at an assisted reproductive unit in patients with recurrent IVF failure to examine their hysteroscopic findings and the effect of their correction on subsequent pregnancy outcomes. Hysteroscopy was done in 157 patients, which showed abnormal findings in 44.9% of the patients, the majority of which were endometrial polyps. Seventy-five women achieved pregnancy following hysteroscopy, with 36 achieving pregnancy after correction of endometrial pathology [11].

A systematic review and meta-analysis showed that inducing mechanical endometrial injury preceding ovarian stimulation for IVF improves implantation rate in women with recurrent IVF failures. The injury was induced via endometrial biopsy/scratch or hysteroscopy. The meta-analysis involved 7 controlled studies (4 randomized and 3 nonrandomized) with an aggregate of 2062 participants and showed that inducing local endometrial injury in the cycle preceding ovarian stimulation is 70% more likely to result in a pregnancy than using no intervention. However, the authors suggested that large randomized studies showed no difference in IVF success after endometrial scratching [12].

It is not clear yet if abnormal hysteroscopic findings, by guiding infertility treatments, increase pregnancy rates. In a study by La Sala *et al.*, hysteroscopy was suggested to be considered as a routine examination in an infertile woman because it would be economically advantageous in regard to costs of assisted reproductive technology [13]. In another study, conducted by Oliveira *et al.* and published in 2003 [14], it was reported that significant unsuspected intrauterine abnormalities were found only with hysteroscopy in 25% of patients with repeated failed IVF and embryo transfer cycles. All the patients in the study had normal HSG in the prior year. More notably, relevant therapeutic interventions significantly improved the pregnancy rate in those with an abnormal uterine cavity on hysteroscopy [14]. Hysteroscopy can therefore diagnose small intrauterine lesions that might affect fertility much more precisely than with HSG and even transvaginal ultrasonography.

## Conclusion

Patients with recurrent IVF embryo transfer failures should be reevaluated using hysteroscopy prior to initiating further IVF embryo transfer cycles in order to increase the clinical pregnancy outcome. Moreover, hysteroscopy should be considered as a crucial component of the initial evaluation of infertile women with recurrent implantation failure. Further studies need to be conducted to establish an explicit connection between hysteroscopic findings and a specified treatment for infertility.

## Data Availability

Yes.

## References

[1] Penzias AS. Recurrent IVF failure: other factors. Fertil Steril. 2012;97(5):1033–8. 10.1016/j.fertnstert.2012.03.017 22464759

[2] Ayida G, Chamberlain P, Barlow D, Kennedy S. Uterine cavity assessment prior to *in vitro* fertilization: comparison of transvaginal scanning, saline contrast hysterosonography and hysteroscopy. Ultrasound Obstet Gynecol. 1997;10:59–62. 10.1046/j.1469-0705.1997.10010059.x 9263425

[3] Demirol A, Gurgan T. Effect of treatment of intrauterine pathologies with office hysteroscopy in patients with recurrent IVF failure. Reprod Biomed Online. 2004;8:590–4. 10.1016/s1472-6483(10)61108-x 15151729

[4] Doldi N, Persico P, Di SF, Marsiglio E, De SL, Rabellotti E, Fusi F, Brigante C, Ferrari A. Pathologic findings in hysteroscopy before *in vitro* fertilization-embryo transfer (IVF-ET). Gynecol Endocrinol. 2005;21:235–7. 10.1080/09513590500366696 16316847

[5] Rama Raju GA, Shashi KG, Krishna KM, Prakash GJ, Madan K. Assessment of uterine cavity by hysteroscopy in assisted reproduction programme and its influence on pregnancy outcome. Arch Gynecol Obstet. 2006;274:160–4. 10.1007/s00404-006-0174-7 16715289

[6] Bettocchi S, Nappi L, Ceci O, Selvaggi L. Office hysteroscopy. Obstet Gynecol Clin North Am. 2004;31:641–54. 10.1016/j.ogc.2004.05.007 15450325

[7] Brown SE, Coddington CC, Schnorr J, Toner JP, Gibbons W, Oehninger S. Evaluation of outpatient hysteroscopy, saline infusion hysterosonography and hysterosalpingography in infertile women: a prospective, randomized study. Fertil Steril. 2000;74:1029–34. 10.1016/s0015-0282(00)01541-7 11056254

[8] Hamou JE. Hysteroscopy and microcolpohysteroscopy: text and atlas. Norwalk: Appleton & Lange; 1991.

[9] Practice Committee of the American Society for Reproductive Medicine. Diagnostic evaluation of the infertile female: a committee opinion. Fertil Steril. 2012;98(2):302–7. 10.1016/j.fertnstert.2012.05.032 22698637

[10] Frydman R, Belaisch-Allart JC. Results of *in vitro* fertilization for endometriosis. Contrib Gynecol Obstet. 1987;16:328–31. 3691102

[11] Cenksoy P, Ficicioglu C, Yıldırım G, Yesiladali M. Hysteroscopic findings in women with recurrent IVF failures and the effect of correction of hysteroscopic findings on subsequent pregnancy rates. Arch Gynecol Obstet. 2013;287(2):357–60. 10.1007/s00404-012-2627-5 23183783

[12] Lensen S, Wilkinson J, Sadler L. A randomized trial of endometrial scratching before *in vitro* fertilization [letter]. N Engl J Med. 2019;380:18. 1777–8 10.1056/NEJMc1902642 31042838

[13] La Sala GB, Montanari R, Dessanti L, Cigarini C, Sartori F. The role of diagnostic hysteroscopy and endometrial biopsy in assisted reproductive technologies. Fertil Steril. 1998;70:378–80. 10.1016/s0015-0282(98)00147-2 9696242

[14] Oliveira FG, Abdelmassih VG, Diamond MP, Dozortsev D, Nagy ZP, Abdelmassih R. Uterine cavity findings and hysteroscopic interventions in patients undergoing *in vitro* fertilization-embryo transfer who repeatedly cannot conceive. Fertil Steril. 2003;80:1371–5. 10.1016/j.fertnstert.2003.05.003 14667871

